# Osteoarthritis of the Knee

**DOI:** 10.1186/1471-2474-16-S1-S8

**Published:** 2015-12-01

**Authors:** Jean McQuade

**Affiliations:** 1 Arthritis Australia

## 

Self-management (SM) programs for people with arthritis or other chronic diseases are commonplace and usually delivered by lay leaders. There is evidence indicating these programs are effective, although systematic reviews show these improvements are small.

The self-management program for Osteoarthritis of the Knee (OAK) differs from other programs in a number of aspects. It is disease-specific and tailored for people with OAK. It was developed using an ENAT survey, a collaborative approach, and a Plan Do Study Act (PDSA) model. It was planned purposely for implementation in hospitals or community settings. The program is detailed for delivery by health professionals. In particular, exercise and disease coping strategies are promoted within a SM construct to improve quality of life and general health as well as reducing pain.

The program was tested using an uncontrolled quality assurance study and the results were positive in pain, quality of life and physical function.

A randomised controlled trial (RCT) was undertaken, where, the OAK group showed statistically significant improvements when compared with the control group with regard to pain, quality of life and function on the basis of WOMAC and SF-36 measurements taken 8 weeks and 6 months from baseline.

The OAK program is conducted in a group setting over six weekly sessions of 2.5 hours. This allows participants to progress over time by incorporating and consolidating new information learned each week. In addition to the weekly sessions, handouts and reference book readings are given.

For optimum group dynamics, the program is delivered by the same two health professionals to a group of 12-14 participants. The fidelity of the OAK program is maintained by the use of a scripted facilitators’ manual.

Using a holistic approach, the program addresses multiple aspects of care: osteoarthritis (explanation and implications), SM skills (goal-setting, problem-solving, positive thinking and improving self-efficacy), medications (types, interactions and current trends), pain management strategies (cognitive and pharmacologic), fitness and exercise (strength, flexibility, aerobic), joint protection, nutrition and weight control, falls prevention (balance and proprioception), environmental risks, and coping skills.

The OAK program has been sustained by development of health professional training workshops. OAK programs are delivered throughout Australia primarily by physiotherapists especially those working in district hospitals or primary care settings. To reduce costs, we have recently trialled programs using self-management trained peer leaders and health professionals which has worked extremely well.

